# An improved predator-prey particle swarm optimization algorithm for Nash equilibrium solution

**DOI:** 10.1371/journal.pone.0260231

**Published:** 2021-11-24

**Authors:** Yufeng Meng, Jianhua He, Shichu Luo, Siqi Tao, Jiancheng Xu

**Affiliations:** 1 School of Electronics and Information, Northwestern Polytechnical University, Xi’an, Shaanxi, China; 2 Southwest China Research Institute of Electronic Equipment, Chengdu, Sichuan, China; Torrens University Australia, AUSTRALIA

## Abstract

Focusing on the problem incurred during particle swarm optimization (PSO) that tends to fall into local optimization when solving Nash equilibrium solutions of games, as well as the problem of slow convergence when solving higher order game pay off matrices, this paper proposes an improved Predator-Prey particle swarm optimization (IPP-PSO) algorithm based on a Predator-Prey particle swarm optimization (PP-PSO) algorithm. First, the convergence of the algorithm is advanced by improving the distribution of the initial predator and prey. By improving the inertia weight of both predator and prey, the problem of “precocity” of the algorithm is improved. By improving the formula used to represent particle velocity, the problems of local optimizations and slowed convergence rates are solved. By increasing pathfinder weight, the diversity of the population is increased, and the global search ability of the algorithm is improved. Then, by solving the Nash equilibrium solution of both a zero-sum game and a non-zero-sum game, the convergence speed and global optimal performance of the original PSO, the PP-PSO and the IPP-PSO are compared. Simulation results demonstrated that the improved Predator-Prey algorithm is convergent and effective. The convergence speed of the IPP-PSO is significantly higher than that of the other two algorithms. In the simulation, the PSO does not converge to the global optimal solution, and PP-PSO approximately converges to the global optimal solution after about 40 iterations, while IPP-PSO approximately converges to the global optimal solution after about 20 iterations. Furthermore, the IPP-PSO is superior to the other two algorithms in terms of global optimization and accuracy.

## Introduction

During the strategy selection for a swarm of Unmanned Aerial Vehicles (UAVs), not only the state of UAV itself but also the strategy of the enemy should be considered. Therefore, more scientific and reasonable strategies can be made by introducing game theory strategies into the confrontation decision of UAVs. Thus, game theory was introduced into UAV confrontation decision-making [1–5]. Due to the large number of swarming UAVs and the many available alternative countermeasures, each strategy needs to be evaluated, resulting in the amount of digital storage space needed for the evaluation values exploding exponentially as the number of countermeasures increases. Therefore, problems such as slow algorithm convergence or convergence to the local optimal solution will be encountered when solving the Nash equilibrium solution of such a game.

At present, the main methods for solving the Nash equilibrium solution include the game solution based on an evolutionary algorithm, an ant colony algorithm, application of graph theory and use of particle swarm optimization (PSO).

Evolutionary Strategies [6–9] are search algorithms proposed by Rechenberg and Schwefel from Germany. In each iteration of the algorithm, each group of individuals (parent generation individuals) in the population recombine and mutate (mutation) to produce offspring individuals. Recombination can be achieved with discrete recombination, median recombination and mixed recombination. Mutation is added by adding a random vector subject to a normal distribution, and the randomness thereof is controlled by manipulating the variance with this variance representing the mutation degree. Using an evolutionary algorithm to solve game problems [10] will use binary encoding strategies for chromosomes, and each player constitutes a chromosome. The situation faced by a player is converted into two binary chromosome groups, using different encoding, crossover, mutation and selection strategies, thus generating new situations through the calculation of fitness functions, and selection of favorable or good chromosomes. In this manner, the Nash equilibrium solution of the game is obtained.

An Ant Colony System is an intelligent biological algorithm proposed by Italian scholars Dorigo, Maniezzo et al. [11], through studying the foraging behavior of ants. An improved ant colony algorithm was proposed for solving Nash equilibrium solutions of finite N-player non-cooperative games [12]. In this class of analysis, ants are first randomly distributed in the range of the feasible solution and initial parameters. Thereafter, based on an individual ant’s variable pheromone strength and fitness function calculation of transition probability, and global search, an ant deploys a dynamic random search technique for local searches, and then updates their pheromone strength. When the optimal solution meeting the precision requirements of the algorithm reaches the maximum allowed number of iterations, the output is considered the optimal solution. Compared with the evolutionary algorithm, the improved ant colony algorithm has better computational performance and can more quickly converge to a more precise solution.

Graph Theory has its roots in the Konigsberg problem. A solution method of Nash equilibrium based on graph theory was proposed to solve two-person non-cooperative pure strategy games [13]. The problem of solving the Nash equilibrium solution of the game model is transformed into the problem of solving the confluence point of the directed graph. If there is a confluence point in the graph, then that point is a Nash equilibrium solution. If there is no confluence point in the graph, then the game has no Nash equilibrium solution. Compared with other methods, the graph theory method finds Nash equilibrium by finding the confluence point, which is more intuitive and simple, and has lower time complexity that other options while improving the speed of solving Nash equilibrium solution of two-person non-cooperative game.

Finally, a PSO method [14, 15] can be used to solve the Nash equilibrium of the attack and defense confrontation game of clustered UAVs.

Aiming at the dynamic multi-strategy of UAV cooperative attack, Wang Y et al. [1] combined the dynamic target assignment problem with the Nash equilibrium concept of game theory, and used the improved PSO algorithm based on an elite reselection mechanism to obtain the optimal strategy combination of both sides in the sense of Nash equilibrium.

A game theory method based on Predator-Prey Particle Swarms Optimization (PP-PSO) was proposed [2], which de-composed the dynamic task assignment problem of multiple UAVs in military operations and modeled it as a two-person game at each decision-making stage. In each decision stage, both parties seek the best solution for the purpose of maximizing their own objective function, and use the PP-PSO algorithm to solve the mixed Nash equilibrium as the optimal allocation scheme at each stage.

The original PSO algorithm easily falls into local optimum. Inspired by the swarm behavior of sardines and herring, this algorithm was optimized according to the predation behavior, and this improved the subject algorithm’s shortcoming regarding its prevalence of local optimization to a certain extent [16–18]. However, due to the use of two populations, the convergence speed of the algorithm is slow when approaching the global optimal value, and the whole population cannot entirely converge to the global optimal value (that is, the average fitness value of the population cannot converge). This paper mainly studies a generally improved Predator-Prey Particle Swarm Optimization(IPP-PSO) algorithm to solve the Nash equilibrium of the game. This algorithm can also be used to solve the Nash equilibrium of the game problem of the attack and defense of UAV swarms.

The main contributions of this paper are summarized as follows:

- Improved initial predator and prey distribution. During initialization, some particles with better fitness values are deemed as prey, and the remaining particles with poorer fitness values are set as predators. The predators can improve the convergence of the algorithm by simultaneously chasing the target and prey;
- Improved inertia weights in predators and prey. In the inertial weight value, the average fitness value of the population of particles is considered, so that the nearer the particle is to the global optimal, the smaller the inertial weight value is, so as to improve the “precocity” problem of the algorithm;
- Improved particle velocity calculation formula. When the fitness value of a particle is less than a certain threshold value, the particle no longer considers its own population optimal, but approaches the global optimal, so as to solve the problems of global optimal and algorithm convergence speed;
- Add pathfinder to increase the diversity of the population. Pathfinder can be a particle randomly distributed in the solution space, or it can be a particle evolved from the current global optimal solution. Pathfinder can improve the global search ability of the algorithm, the “precocity” of the better algorithm and the problem of the global optimal solution.

This paper is organized as follows: Section I introduces the reason why we establish the IPP-PSO. Section II introduces the related word of the PSO. Section III introduces the PSO and PP-PSO. In section IV the IPP-PSO algorithm is established. Section V shows the simulation results and analysis. Finally, conclusions are set forth in Section VI.

## Related work

The implementation process of the PSO algorithm has a great relationship with the value of its parameters. How to select these parameters is an urgent problem to be solved. When a PSO algorithm is applied to the optimization of complex high-dimensional problems, premature convergence and other problems are often encountered [19]. In the past two decades, a large number of scholars have conducted in-depth research on the improvement of the PSO algorithm.

The theoretical research of the PSO algorithm includes improved strategy research of PSO algorithm. Among them, the theoretical study of PSO algorithms focuses on the dynamic characteristics of such algorithms and the topological structure of PSO algorithm. Furthermore, the research studies the control parameters of PSO algorithms. The improved strategy deployed therein mainly focused on adaptive PSO control parameters, improved learning strategies, and mixed PSO strategies [20].

The problems of the multi-objective PSO algorithm are as follows:

1) How to select “leader” particles in the optimization process to lead the entire population to quickly approach the Pareto front under the premise of retaining some individual information, that is, the optimal particle selection strategy;
2) In PSO, the population individuals are affected by the “optimal” particles, and the rapid convergence leads to “precocity”. This then presents the question of how to guide the particles to “jump out” of the local optimal, that is, the diversity preservation mechanism;
3) As the number of non-dominated solutions in the external storage concentration increases rapidly, then how to guide the population to further improve the search efficiency under the premise of ensuring the diversity of the population, so as to strengthen the advantages of the algorithm in the convergence rate, that is, the means to improve the convergence;
4) How to dynamically coordinate the relationship between the whole development and local search in different stages of the optimization process to obtain the best optimization results, that is, the balance method of diversity and convergence.

In order to improve the performance of the algorithm, iterative formula improvement, dynamic tuning of important parameters and adjustment of information interaction between particles are carried out, that is, the iterative formula, parameters and topology improvement scheme [21]. The algorithm improvement measures for these problems include: the selection of optimal particles, the preservation of diversity, the improvement of convergence, the balance of diversity and convergence, and the improvement of iterative formula, parameters and topology structure.

As a group optimization algorithm, in order to improve the search ability and avoid falling into local optimal, the PSO should maintain diversity in the early stage of the algorithm. In the later stages of the algorithm, it is particularly important to improve its convergence ability [22]. Common diversity control methods are as follows: diversity control based on particle spacing, diversity control based on exclusion and disturbance, diversity control based on multiple subgroups, and population diversity detection.

The improvement measures of the PSO algorithm are as follows [23]: 1) Increase the inertia weight and convergence factor to improve the convergence speed; 2) PSO with a selection mechanism will inhibit the control effect of a few super particles and improves the success rate of convergence; 3) PSO with a mutation operator can adjust the search speed of particles and improve the convergence speed and accuracy; 4) Based on the improvement of neighborhood operators and topology structure, different neighborhood topologies are used to study the performance of guaranteed convergence PSO; 5) A new particle structure or group structure is constructed to effectively improve the global convergence ability of the algorithm; 6) Improve or use the new position/speed update formula and improve the global and local search ability, thereby avoiding the algorithm falling into the local optimal; 7) The organic combination of other evolutionary optimization technology PSO algorithm.

## Two traditional particle swarm optimization algorithms

### Particle swarm optimization

PSO is an algorithm that simulates the foraging behavior of birds. If a flock of birds in a random search of their area for food, and all the birds do not know the exact location of food, but they know that others in their location will be searching together for the flock to find the nearest food, then during the process of search and to avoid any adjacent bird collision, they match both the surrounding birds’ speed, and their own vector to the target.

**Fig 1:**
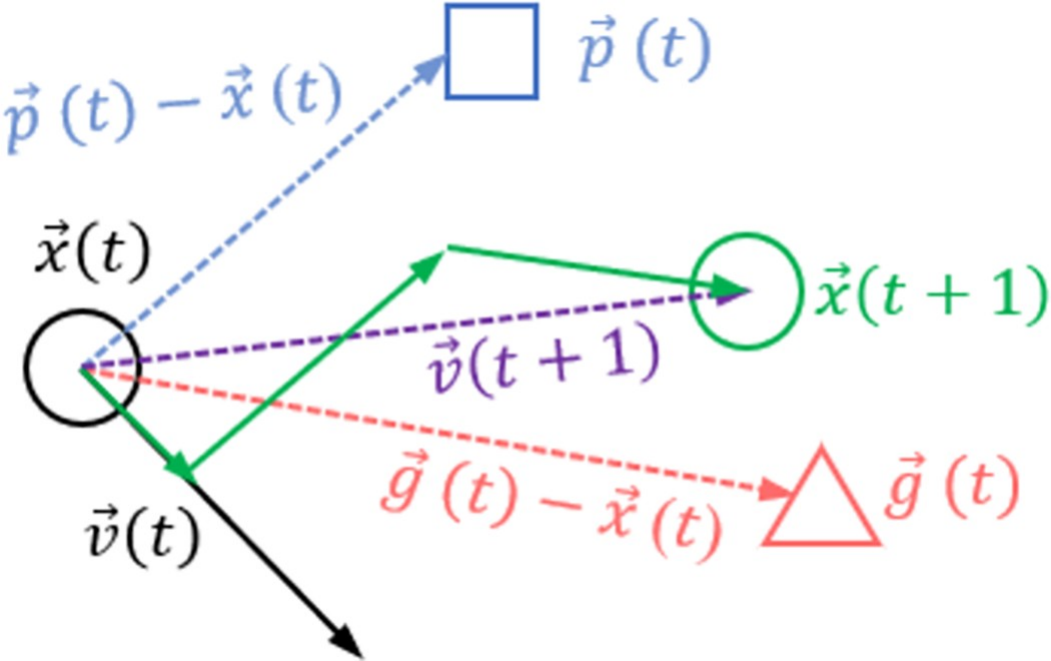
Particle flight velocity diagram.

The foraging behavior of birds is the origin of the idea of the PSO algorithm. A particle is used to represent a bird, then the current position of each particle is a feasible solution of the problem to be solved. The particle searches in the n-dimensional space, and the search process of the particle is factored into the flight process. The velocity is adjusted according to the historical optimal solution of the particle itself and the optimal solution of the population. The adjustment method is shown in Fig 1:

In Fig 1, $\vec{x}\left(t\right)$ is the position of the particle at time t, $\vec{x}\left(t+1\right)$ is the position of the particle at the next unit of time (time t+1), $\vec{v}\left(t\right)$ is the velocity of the particle at time t, $\vec{v}\left(t+1\right)$ is the velocity of the particle at time t, $\vec{p}\left(t\right)$ is the optimal solution of the particle before time t, and $\vec{g}\left(t\right)$ is the historical optimal solution of the whole particle swarm. Therefore, the formula for particle I to update its own velocity and position is shown in (1) and (2):

$$\begin{array}{c} v_{i}\left(t+1\right)=w\times v_{i}\left(t\right)+c_{1}r_{1}\left(p_{i}\left(t\right)-x_{i}\left(t\right)\right)+c_{2}r_{2}\left(g\left(t\right)-x_{i}\left(t\right)\right) \end{array}$$ (1)

$$\begin{array}{c} x_{i}\left(t+1\right)=x_{i}\left(t\right)+v_{i}\left(t\right) \end{array}$$ (2)

In (1), *w* is the inertia factor; *c*_1_,*c*_2_ are learning factors, also known as the acceleration constants; and *r*_1_,*r*_2_ are each a uniform random number within the range [0, 1].

It is assumed that the feasible solution space is D- dimensional, and the particle swarm consists of N particles. The relevant symbols are as follows:

Position of the *i*^*th*^ particle:

$$\begin{array}{c} X_{i}=\left(x_{i1},x_{i2},\ldots ,x_{iD}\right),i=1,2,\ldots ,N \end{array}$$ (3)

The flight speed of the *i*^*th*^ particle:

$$\begin{array}{c} V_{i}=\left(v_{i1},v_{i2},\ldots ,v_{iD}\right),i=1,2,\ldots ,N \end{array}$$ (4)

The historical optimal solution of the *i*^*th*^ particle is the individual extreme value:

$$\begin{array}{c} P_{best}^{i}=\left(p_{i1},p_{i2},\ldots ,p_{iD}\right),i=1,2,\ldots ,N \end{array}$$ (5)

The historical optimal solution of the entire particle swarm is the global extremum:

$$\begin{array}{c} g_{best}=\left(p_{g1},p_{g2},\ldots ,p_{gD}\right) \end{array}$$ (6)

The degree of the solution represented by the particle is judged according to the fitness function value.

The steps of the PSO algorithm are as follows:

Step 1: Initialization of the population, including size N of the particle swarm, position *X*_*i*_ and velocity *V*_*i*_ of each particle;
Step 2: Set the maximum number of iterations *CycleMax*, and make the current number of iterations *t* = 1;
Step 3: Calculate the fitness value *F*_*i*_(*t*) of each particle;
Step 4: Compare the fitness value *F*_*i*_(*t*) of each particle with the fitness value $F_{pbest}^{i}$ of pbest $P_{best}^{i}$. If $F_{i}\left(t\right)<F_{pbest}^{i}$, replace $P_{best}^{i}$ with *X*_*i*_(*t*) and $F_{pbest}^{i}$ with *F*_*i*_(*t*).
Step 5: Compare the fitness value *F*_*i*_(*t*) of each particle with the fitness value $F_{gbest}^{i}$ of $g_{best}^{i}$. If *F*_*i*_(*t*)<*F*_*gbest*_, replace *g*_*best*_ with *X*_*i*_(*t*) and *F*_*gbest*_ with *F*_*i*_(*t*).
Step 6: Update the velocity vector of each particle according to (1);
Step 7: Update the position vector of each particle according to (1);
Step 8: Judge whether the maximum number of iterations *CycleMax* or the accuracy requirements are reached. If yes, the simulation ends. Otherwise, return to Step 3.

**Fig 2:**
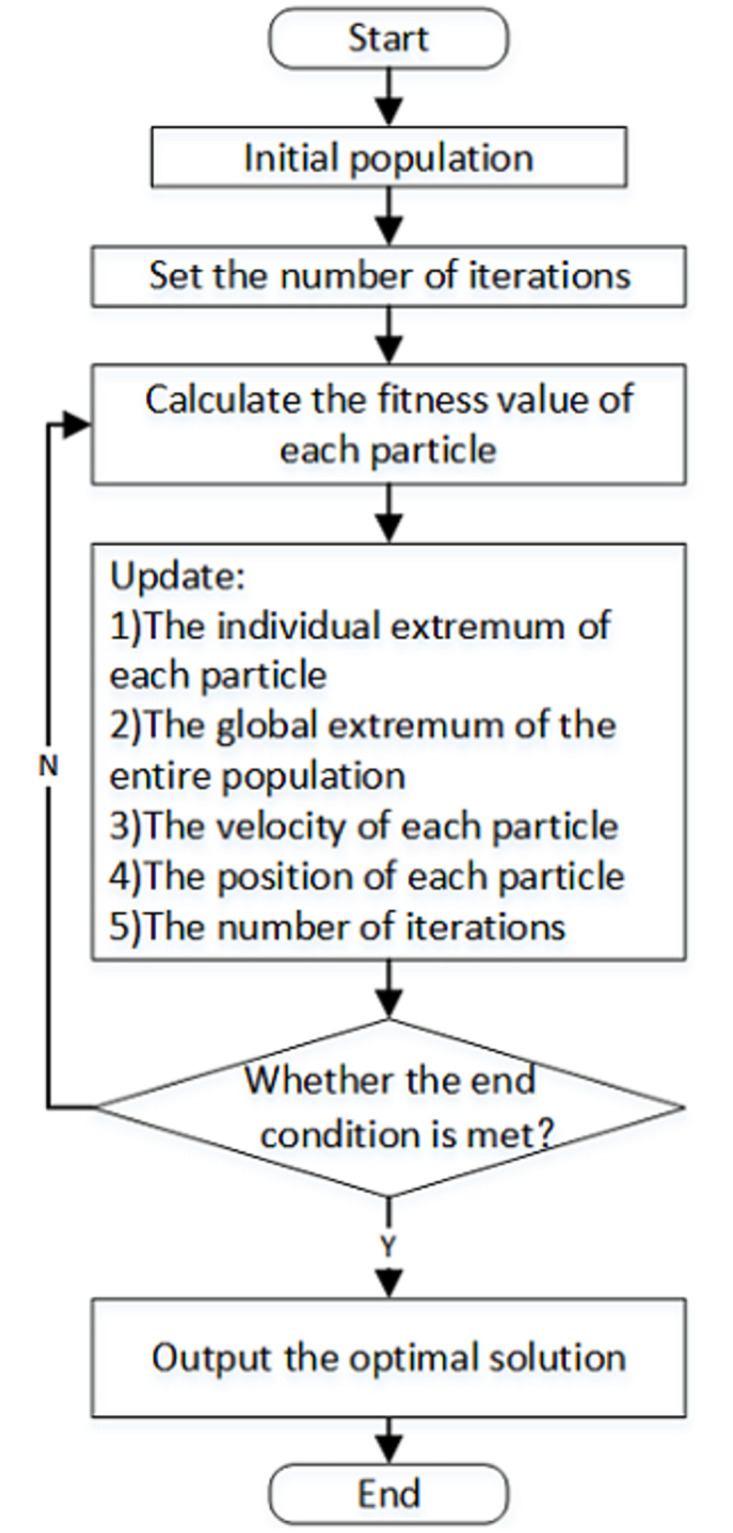
Flow chart of the particle swarm optimization.

The flow chart of particle swarm optimization algorithm is shown in Fig 2.

### Predator-prey particle swarm optimization

Because the original PSO algorithm easily falls into the local optimum, the PSO algorithm was optimized according to the predation behavior [16–18]. This optimization idea was was inspired by the swarm behavior of sardines and herring. Particles are divided into two categories, predator and prey. Predators (particles that prey on) pursue their prey and move toward the center of the prey group; The prey (the escaping particle) escapes from the predator within the range of feasible solutions, and the prey adopt different escape behaviors by weighing the predator risk and energy. The velocity and position of predator and prey in the particle swarm optimization algorithm are defined as (7) and (8):

$$\begin{array}{c} \begin{array}{cc} v_{di}\left(t+1\right)= & w_{d}v_{di}\left(t\right)+c_{1}r_{1}\left(p_{di}\left(t\right)-x_{di}\left(t\right)\right)+\\ & c_{2}r_{2}\left(g_{d}\left(t\right)-x_{di}\left(t\right)\right)+c_{3}r_{3}\left(g\left(t\right)-x_{di}\left(t\right)\right) \end{array} \end{array}$$ (7)

$$\begin{array}{c} \begin{array}{cc} v_{ri}\left(t+1\right)= & w_{r}v_{ri}\left(t\right)+c_{4}r_{4}\left(p_{ri}\left(t\right)-x_{ri}\left(t\right)\right)+c_{5}r_{5}\left(g_{r}\left(t\right)-x_{ri}\left(t\right)\right)+c_{6}r_{6}\left(g\left(t\right)-x_{ri}\left(t\right)\right)\\ & -P\cdot a\cdot sign\left[x_{dI}\left(t\right)-x_{ri}\left(t\right)\right]\cdot \;\text{exp}\left(-b|x_{dI}\left(t\right)-x_{ri}\left(t\right)|\right) \end{array} \end{array}$$ (8)

$$\begin{array}{c} x_{di}\left(t+1\right)=x_{di}+v_{di}\left(t+1\right) \end{array}$$ (9)

$$\begin{array}{c} x_{ri}\left(t+1\right)=x_{ri}+v_{ri}\left(t+1\right) \end{array}$$ (10)

The *d* and *r* in the subscripts of (6) and (7) denote predator and prey, respectively.

*p*_*di*_ is the historical best solution for the *i*^*th*^ predator (individual extremum), *p*_*ri*_ is the historical best location for the *i*^*th*^ prey, *g*_*d*_ is the historical best solution of the predator population (population extremum), *g*_*r*_ is the historical best solution of prey population (population extremum), *g* is the best position (global extremum) found in the whole particle swarm so far. The definitions of *w*_*d*_ and *w*_*r*_ are shown in Eqs (11) and (12).

$$\begin{array}{c} w_{d}=0.2\;\text{exp}\left(-10\frac{iteration}{iteration_{max}}\right)+0.4 \end{array}$$ (11)

$$\begin{array}{c} w_{r}=w_{max}-\frac{w_{max}-w_{min}}{iteration_{max}}iteration \end{array}$$ (12)

*w*_*d*_ and *w*_*r*_ are the inertia weights of predator and prey, respectively. The value that inertia weights plays an important role in the global and local search ability and algorithm convergence of the algorithm. *iteration*_*max*_ represents the maximum number of iterations. *w*_*max*_ and *w*_*min*_ represent the maximum and minimum values of *w*_*r*_, respectively. The definition of *I* in (8) is shown in (13)

$$\begin{array}{c} I=\left\{k\right|\underset{k}{min}\left(\right|x_{dk}-x_{ri}\left|)\right\} \end{array}$$ (13)

*I* is the number of predators around the *i*^*th*^ prey. In (8), *P* indicates whether the prey is able to escape (*P* = 0(yes) or *P* = 1 (no)), and *a* and *b* are the parameters that determine how difficult it is for the prey to escape the predator. The closer the predator is to the prey, the harder it is for the prey to escape the predator. *sign* is a symbolic function, defined as Formula (14):

$$\begin{array}{c} sign\left(x\right)=\{\begin{array}{cc} -1 & x<0\\ 0 & x=0\\ 1 & x>0 \end{array} \end{array}$$ (14)

**Fig 3:**
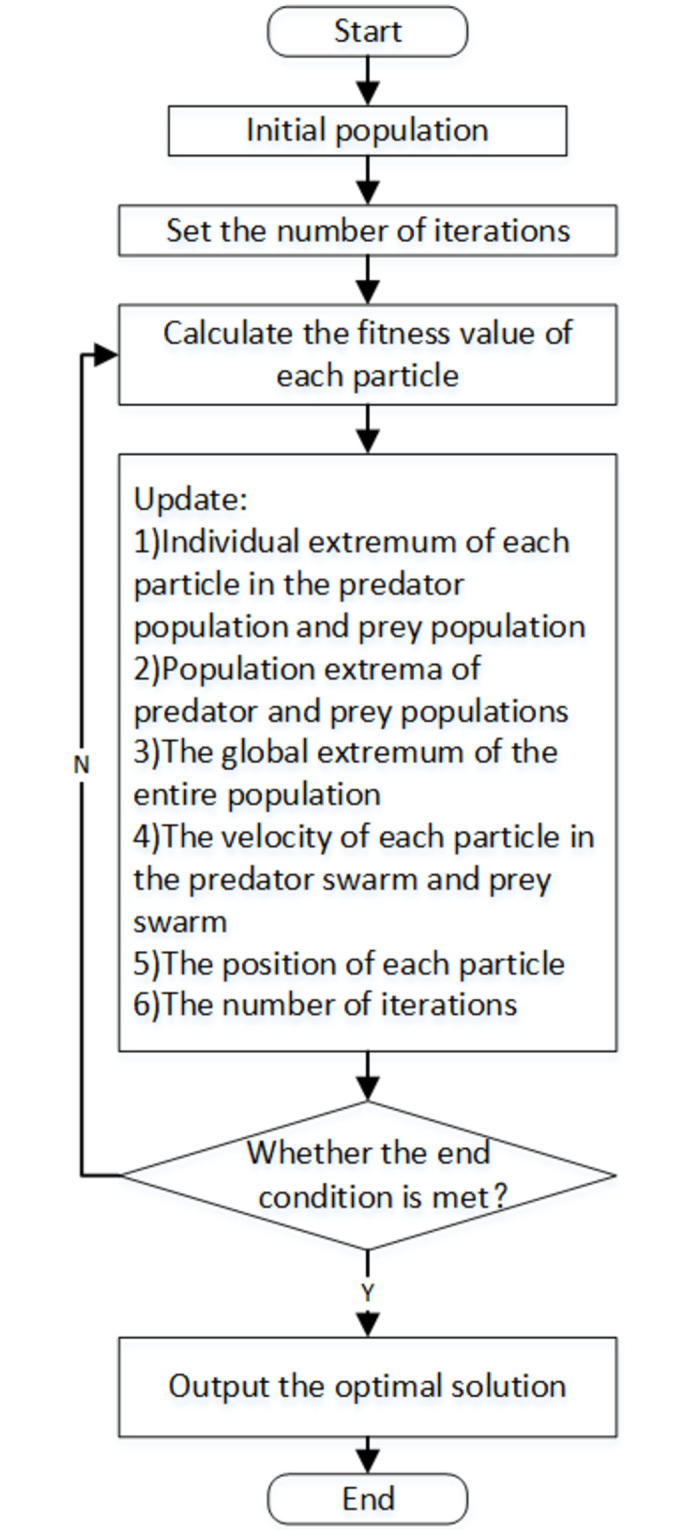
Flow chart of predator-prey particle swarm optimization.

Then the flow chart of Predator-Prey Particle Swarms Algorithm is shown in Fig 3.

The algorithm steps are as follows:

Step 1: Initialization of the population. Set particle swarm size *N*, predator group size *D*, prey group size *R* to satisfy *N* = *D* + *R*, the initial position and initial velocity of each particle;
Step 2: Set the maximum number of iterations to *iteration*_*max*_, and set the current number of iterations to *iteration* = 1;
Step 3: Calculate the fitness values *F*_*di*_(*t*) and *F*_*ri*_(*t*) of each particle in predator group and prey group respectively;
Step 4: Compare the fitness value *F*_*di*_(*t*) of each particle in the predator swarm with the fitness value $F_{pbest}^{di}$ of the individual extreme value $P_{di}^{best}$. If $F_{di}\left(t\right)<F_{pbest}^{di}$, $P_{di}^{best}$ will be replaced by *X*_*di*_(*t*), and $F_{pbest}^{di}$ will be replaced by *F*_*di*_(*t*).

The fitness value *F*_*ri*_(*t*) of each particle in the prey group was compared with the fitness value $F_{pbest}^{ri}$ of the individual extreme value $P_{ri}^{best}$. If $F_{ri}\left(t\right)<F_{pbest}^{ri}$, $P_{ri}^{best}$ was replaced with *X*_*ri*_(*t*), and $F_{pbest}^{ri}$ was replaced with *F*_*ri*_(*t*).

Step 5: Compare the fitness value *F*_*di*_(*t*) of each particle in the predator population with the fitness value $F_{pbest}^{d}$ of the extreme population *g*_*d*_ of the predator population. If $F_{di}\left(t\right)<F_{pbest}^{d}$, then replace *g*_*d*_ with *X*_*di*_(*t*) and $F_{pbest}^{d}$ with *F*_*di*_(*t*).

The fitness value *F*_*ri*_(*t*) of each particle in the prey group was compared with the fitness value $F_{pbest}^{r}$ of the population extreme value *g*_*r*_ of the prey group. If $F_{ri}\left(t\right)<F_{pbest}^{r}$, *g*_*r*_ was replaced by *X*_*ri*_(*t*), and $F_{pbest}^{r}$ was replaced by *F*_*ri*_(*t*).

Step 6: Compare the fitness value *F*_*i*_(*t*) of each particle in the population with the fitness value *F*_*gbest*_ of the global extreme value *g*_*best*_. If *F*_*i*_(*t*) < *F*_*gbest*_, replace *g*_*best*_ with *X*_*i*_(*t*) and *F*_*gbest*_ with *F*_*i*_(*t*).
Step 7: Update the velocity vector of each particle in the predator swarm according to (7);
Step 8: Update the velocity vector of each particle in the prey group according to (8);
Step 9: Update the position vectors of each particle in the predator group and prey group according to (9) and (10) respectively;
Step 10: Determine whether the maximum number of iterations is *iteration*_*max*_ or the accuracy requirements are met. If yes, output the optimal solution and end; otherwise, return to Step 3.

## Improved predator-prey particle swarm optimization

When two populations are used, the convergence speed of the algorithm is slow as it approaches the global optimal value, and the whole population cannot all converge to the global optimal value (that is, the average fitness value of the population cannot converge). According to the above shortcomings, the PP-PSO algorithm is improved as follows:

1) Improved initial predator and prey distribution. For the randomly distributed particles in the solution space, because predators can hunt prey close to the target, during initialization one calculates the population fitness function value for all the particles. The fitness value is set higher for prey particles, with the remainder of the poor fitness values set as predators, and predators (by chasing targets at the same time) and prey improve the convergence of the algorithm.

2) Improved inertia weights in predators and prey. In the PP-PSO algorithm, the inertia weight is related to the number of iterations. With an increase in the number of iterations, the inertia weight gradually decreases. However, the increase of the number of iterations does not mean that the algorithm has converged to the global optimal. Therefore, the average fitness value of the population particle is considered in the inertia weight value, that is, the closer the particle is to the global optimal, the smaller the inertia weight is, thus improving the problem of “precocity” of the algorithm. The improved definitions of *w*_*d*_ and *w*_*r*_ are shown in (15) and (16):

$$\begin{array}{c} w_{d}=\left(0.2\;\text{exp}\left(-10\frac{iteration}{iteration_{max}}\right)+0.4\right)\cdot \left(1-\text{exp}(\frac{-1}{D}\sum_{i=1}^{D}F_{di}\left(t\right)\right)) \end{array}$$ (15)

$$\begin{array}{c} w_{r}=\left(w_{max}-\frac{w_{max}-w_{min}}{iteration_{max}}iteration\right)\cdot \left(1-\text{exp}(\frac{-1}{R}\sum_{i=1}^{R}F_{ri}\left(t\right)\right)) \end{array}$$ (16)

3) Improved particle velocity calculation formula. In the PP-PSO algorithm, the velocity formulas of predator and prey particles do not change in the iterative process. As the predator population is affected by the optimal solution of prey population and its own population, the whole population cannot converge to the global optimal value. According to this shortcoming, the velocity calculation formula of the particle is improved. When the fitness value of the particle is less than a certain threshold, the particle no longer considers the population optimization and approaches the global optimization. The threshold can be determined by Monte Carlo simulation thusly: Within the range of the fitness function, *p* as the step length, generate *q* threshold points (*q* = the range divided by *p*, round operation); The threshold points are obtained with equal probability, and simulation is performed every time a threshold point is obtained; The simulation runs *n* times; The number of iterations of global optimization calculated for the same threshold point is statistically processed, and the threshold point with the best performance is selected as the final threshold point of the entire algorithm. Then, the velocity definitions of predator and prey in the improved predator-prey particle swarm optimization algorithm are shown in Eqs (17) and (18):

$$\begin{array}{c} \begin{array}{cc} & v_{di}\left(t+1\right)=\\ & \left\{ \begin{array}{c} \begin{array}{ccc} & w_{d}v_{di}\left(t\right)+c_{1}r_{1}\left(p_{di}\left(t\right)-x_{di}\left(t\right)\right)+c_{2}r_{2}\left(g_{d}\left(t\right)-x_{di}\left(t\right)\right)+\\ & c_{3}r_{3}\left(g_{r}\left(t\right)-x_{di}\left(t\right)\right)+c_{4}r_{4}\left(g\left(t\right)-x_{di}\left(t\right)\right) & F_{di}\left(t\right)>threshold\\ & w_{d}v_{di}\left(t\right)+c_{1}r_{1}\left(p_{di}\left(t\right)-x_{di}\left(t\right)\right)+c_{3}r_{3}\left(g\left(t\right)-x_{di}\left(t\right)\right) & F_{di}\left(t\right)\leq threshold \end{array} \end{array}\right. \end{array} \end{array}$$ (17)

$$\begin{array}{c} \begin{array}{cc} & v_{ri}\left(t+1\right)=\\ & \left\{ \begin{array}{c} \begin{array}{ccc} & w_{r}v_{ri}\left(t\right)+c_{5}r_{5}\left(p_{ri}\left(t\right)-x_{ri}\left(t\right)\right)+c_{6}r_{7}\left(g_{r}\left(t\right)-x_{ri}\left(t\right)\right)+\\ & c_{7}r_{7}\left(g_{r}\left(t\right)-x_{ri}\left(t\right)\right) & F_{ri}\left(t\right)>threshold\\ & w_{r}v_{ri}\left(t\right)+c_{5}r_{5}\left(p_{ri}\left(t\right)-x_{ri}\left(t\right)\right)+c_{7}r_{7}\left(g\left(t\right)-x_{ri}\left(t\right)\right) & F_{ri}\left(t\right)\leq threshold \end{array} \end{array}\right. \end{array} \end{array}$$ (18)

4) Add pathfinder to increase the diversity of the population. Through improving the distribution of the initial predator and prey inertia weight values while improving the particle velocity formula we can improve the convergence speed of algorithm in theory, but it may make the algorithm demonstrate a “premature” phenomenon. Therefore, introducing the pathfinder to a random distribution in the solution space of particles can also be used for the current global optimal solutions evolved particles. In this manner, the global searching ability of the algorithm is improved by means of pathfinder. According to the algorithm principle, the optimization degree of the individual extremum *δ*_*p*_(*t*) and the global extremum *δ*_*g*_(*t*) in the t iteration can be obtained, as shown in (19) and (20):

$$\begin{array}{c} \delta_{p}\left(t\right)=\sum_{i=1}^{D}F_{di}\left(t\right)+\sum_{i=1}^{R}F_{ri}\left(t\right)-\left(\sum_{i=1}^{D}F_{di}\left(t-1\right)+\sum_{i=1}^{R}F_{ri}\left(t-1\right)\right) \end{array}$$ (19)

$$\begin{array}{c} \delta_{g}\left(t\right)=F_{gbest}\left(t\right)-F_{gbest}\left(t-1\right) \end{array}$$ (20)

If the individual optimization threshold *OptimalValue* is set, when

$$\begin{array}{c} \delta_{p}\left(t\right)<OptimalValue\times N \end{array}$$ (21)

and

$$\begin{array}{c} \delta_{g}\left(t\right)<OptimalValue \end{array}$$ (22)

This then indicates that the optimization degree of the algorithm in the t iteration is low. Therefore, the pathfinder is added, and the pathfinder can evolve a new solution in the feasible solution space according to the current global extreme value, or generate a new solution randomly in the feasible solution space. Pathfinder evolves according to the evolutionary formula (23) of the evolutionary strategy:

$$\begin{array}{c} X_{ti}^{k}\left(t\right)=g^{k}\left(t\right)+N\left(0,\sigma \right) \end{array}$$ (23)

In (23), $X_{ti}^{k}\left(t\right)$ is the *k*^*th*^ component of the *i*^*th*^ pathfinder particle; *g*^*k*^(*t*) is the *k*^*th*^ component of the global extremum; and *N*(0, *σ*) is a random number following a normal distribution, with a mean of zero and a standard deviation of *σ*.

**Fig 4:**
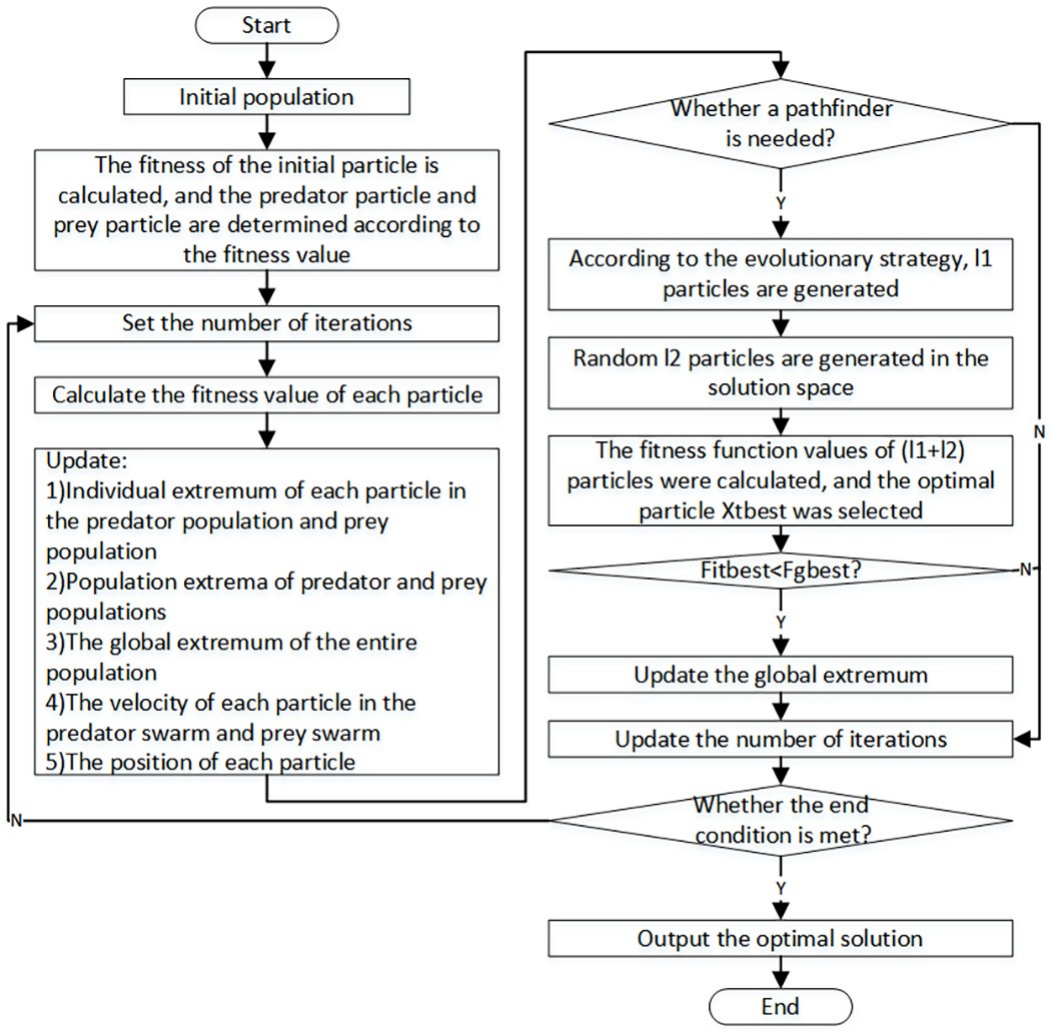
Improved predator-prey particle swarm optimization flow chart.

According to the above improvements, the flow chart of the improved predator-prey particle swarm optimization is shown in Fig 4. The steps of the algorithm are as follows:

Step 1: Initialization of the population. Set particle swarm size *N*, predator group size *D*, prey group size *R* to satisfy *N* = *D* + *R*, giving the initial position and initial velocity of each particle.
Step 2: Calculate the fitness value *F*_*i*_(0) of each initial particle, and sort the fitness value from small to large. The particle corresponding to the fitness value of the first ranked *R* is prey, and the remaining particles are predators.
Step 3: Set the maximum number of iterations *iteration*_*max*_, and set the current number of iterations to *iteration* = 1;
Step 4: Calculate the fitness values *F*_*di*_(*t*) and *F*_*ri*_(*t*) of each particle in predator group and prey group respectively;
Step 5: Compare the fitness value *F*_*di*_(*t*) of each particle in the predator group with the fitness value $F_{pbest}^{di}$ of individual extreme value $P_{di}^{best}$, if $F_{di}\left(t\right)<F_{pbest}^{di}$, replace $P_{di}^{best}$ with *X*_*di*_(*t*), and $F_{pbest}^{di}$ with *F*_*di*_(*t*);

By comparing the fitness value *F*_*ri*_(*t*) of each particle in the prey group with the fitness value $F_{pbest}^{ri}$ of individual extreme value $P_{ri}^{best}$, if $F_{ri}\left(t\right)<F_{pbest}^{ri}$, replace $P_{ri}^{best}$ with *X*_*ri*_(*t*), and $F_{pbest}^{ri}$ with *F*_*ri*_(*t*);

Step 6: Compare the fitness value *F*_*di*_(*t*) of each particle in the predator population with the fitness value $F_{pbest}^{d}$ of the population extreme value *g*_*d*_ of the predator population, if $F_{di}\left(t\right)<F_{pbest}^{d}$, replace *g*_*d*_ with *X*_*di*_(*t*), and $F_{pbest}^{d}$ with *F*_*di*_(*t*);

The fitness value *F*_*ri*_(*t*) of each particle in the prey group is compared with the fitness value $F_{pbest}^{r}$ of the population extremum *g*_*r*_ of the prey group, if $F_{ri}\left(t\right)<F_{pbest}^{r}$, replace *g*_*r*_ with *X*_*ri*_(*t*), and $F_{pbest}^{r}$ with *F*_*ri*_(*t*);

Step 7: Compare the fitness value *F*_*i*_(*t*) of each particle in the population with the fitness value *F*_*gbest*_ of the global extreme value *g*_*best*_, if *F*_*i*_(*t*) < *F*_*pbest*_, replace *g*_*best*_ with *X*_*i*_(*t*), and *F*_*pbest*_ with *F*_*i*_(*t*);
Step 8: Update the velocity vector of each particle in the predator swarm according to (17);
Step 9: Update the velocity vector of each particle in the prey group according to (18);
Step 10: Update the position vectors of each particle in the predator group and prey group according to (9) and (10) respectively;
Step 11: Determine if Pathfinder added is needed. If “No”, go to Step 12. If “yes”, *l*_1_ pathfinders are generated according to Formula (17), *l*_2_ pathfinders are randomly generated in the solution space, and fitness function values of *l*_1_ + *l*_2_ pathfinders are calculated, among which the optimal individual *X*_*tbest*_ is selected and compared with the global extremum. If it is better than the global extremum, *g*_*best*_ is replaced by *X*_*tbest*_, and *F*_*gbest*_ is replaced by *F*_*tbest*_.
Step 12: Judge whether the maximum number of iterations *iteration*_*max*_ or the accuracy requirements are met. If “yes”, the optimal solution will be the output and the end will be concluded. If “No”, go back to Step 3.

## Simulation and analysis

The Nash equilibrium solution of the two-player non-cooperative game must be solved, and the payment matrix of both players needs to be calculated by the objective function [24–26]. The payoff matrix for player 1 is *MA*_*m*×*n*_. The payoff matrix for Player 2 is *MB*_*m*×*n*_. *m* is the number of pure strategies for Player 1, and *n* is the number of pure strategies for player 2. Set the mixed strategy vector of both players as:

$$\begin{array}{c} X_{i}=\left(x_{i1},\cdots ,x_{im},y_{i1},\cdots ,y_{in}\right) \end{array}$$ (24)

In (24),

$$\begin{array}{c} x_{ik}\geq 0 \end{array}$$ (25)

$$\begin{array}{c} y_{ik}\geq 0 \end{array}$$ (26)

$$\begin{array}{c} \sum_{k=1}^{m}x_{ik}=1 \end{array}$$ (27)

$$\begin{array}{c} \sum_{k=1}^{n}y_{ik}=1 \end{array}$$ (28)

Therefore, for any mixed strategy *X*_*i*_, the Nash equilibrium solution (X*, Y*) must satisfy the formula:

$$\begin{array}{c} \left\{ \begin{array}{c} MA{\left(Y^{*}\right)}^{\prime }\geq MA{\left(X_{i}\left(m+1:m+n\right)\right)}^{\prime }\\ X^{*}MB\geq X_{i}\left(1:m\right)MB \end{array}\right. \end{array}$$ (29)

Each particle is represented by a mixed strategy of all players in IPP-PSO algorithm. To solve the Nash equilibrium, the fitness function is defined as shown in formula (30) and formula (31):

$$\begin{array}{c} \begin{array}{cc} f[X_{di}^{t}]= & max\{\underset{1\leq j\leq m}{max}\left\{MA\left(j,:\right)\times {\left(X_{di,m+1:m+n}^{t}\right)}^{\prime }-X_{di,1:m}^{t}\times MA\times {\left(X_{di,m+1:m+n}^{t}\right)}^{\prime }\right\},0\}\\ & +max\{\underset{1\leq j\leq n}{max}\left\{X_{di,1:m}^{t}\times MB{\left(:,j\right)}^{\prime }-X_{di,1:m}^{t}\times MB\times {\left(X_{di,m+1:m+n}^{t}\right)}^{\prime }\right\},0\} \end{array} \end{array}$$ (30)

$$\begin{array}{c} \begin{array}{cc} f[X_{ri}^{t}]= & max\{\underset{1\leq j\leq m}{max}\left\{MA\left(j,:\right)\times {\left(X_{ri,m+1:m+n}^{t}\right)}^{\prime }-X_{ri,1:m}^{t}\times MA\times {\left(X_{ri,m+1:m+n}^{t}\right)}^{\prime }\right\},0\}\\ & +max\{\underset{1\leq j\leq n}{max}\left\{X_{ri,1:m}^{t}\times MB{\left(:,j\right)}^{\prime }-X_{ri,1:m}^{t}\times MB\times {\left(X_{ri,m+1:m+n}^{t}\right)}^{\prime }\right\},0\} \end{array} \end{array}$$ (31)

The above fitness function derivation can be found in Jia W et al [27] and Duan H et al [2]. $X_{di,1:m}^{t}$ and $X_{di,m+1:m+n}^{t}$ represent the mixed strategy of player 1 and player 2 in predator *i* respectively in (30).$X_{ri,1:m}^{t}$ and $X_{ri,m+1:m+n}^{t}$ represent the mixed strategy of player 1 and player 2 in prey *i* respectively in (31). The constraint conditions of the mixed strategy are shown in formula (32) and (33):

$$\begin{array}{c} x_{di,j}\left(t\right)\geq 0,x_{ri,j}\left(t\right)\geq 0 \end{array}$$ (32)

$$\begin{array}{c} \left\{ \begin{array}{cc} & \sum_{j=1}^{m}x_{di,j}=1,\sum_{j=m+1}^{m+n}x_{di,j}=1\\ & \sum_{j=1}^{m}x_{ri,j}=1,\sum_{j=m+1}^{m+n}x_{ri,j}=1 \end{array}\right. \end{array}$$ (33)

In order to verify the effectiveness of the proposed algorithm, the performance results of PSO, PP-PSO and IPP-PSO are compared by solving the Nash equilibrium solution of a zero-sum game and a non-zero-sum game.

The relevant initial parameters of the algorithm are set: the maximum number of iterations *iteration*_*max*_ = 100, particle swarm size *N* = 30, inertia weight *w*_*max*_ = 0.9, *w*_*min*_ = 0.2. In the PP-PSO and IPP-PSO algorithms, the population sizes of predators and prey are respectively *D* = 20 and *R* = 10, while in IPP-PSO algorithm, pathfinder *l*_1_ = 15 and *l*_2_ = 15.

The payment function of the two-person zero-sum game and non-zero-sum game [28] are shown in Tables 1 and 2 respectively:

**Table 1:** Two-person zero-sum game payment function.

	palyer2 strategy1	palyer2 strategy2	palyer2 strategy3
palyer1 strategy1	(8,-8)	(9,-9)	(3,-3)
palyer1 strategy2	(2,-2)	(5,-5)	(6,-6)
palyer1 strategy3	(4,-4)	(1,-1)	(7,-7)

**Table 2:** Two-person zero-sum game payment function.

	palyer2 strategy1	palyer2 strategy2	palyer2 strategy3	palyer2 strategy4
palyer1 strategy1	(1,1)	(235,0)	(0,235)	(0.1,1.1)
palyer1 strategy2	(0,235)	(1,1)	(235,0)	(0.1,1.1)
palyer1 strategy3	(235,0)	(0,235)	(1,1)	(0.1,1.1)
palyer1 strategy4	(1.1,0.1)	(1.1,0.1)	(1.1,0.1)	(0,0)

**Fig 5:**
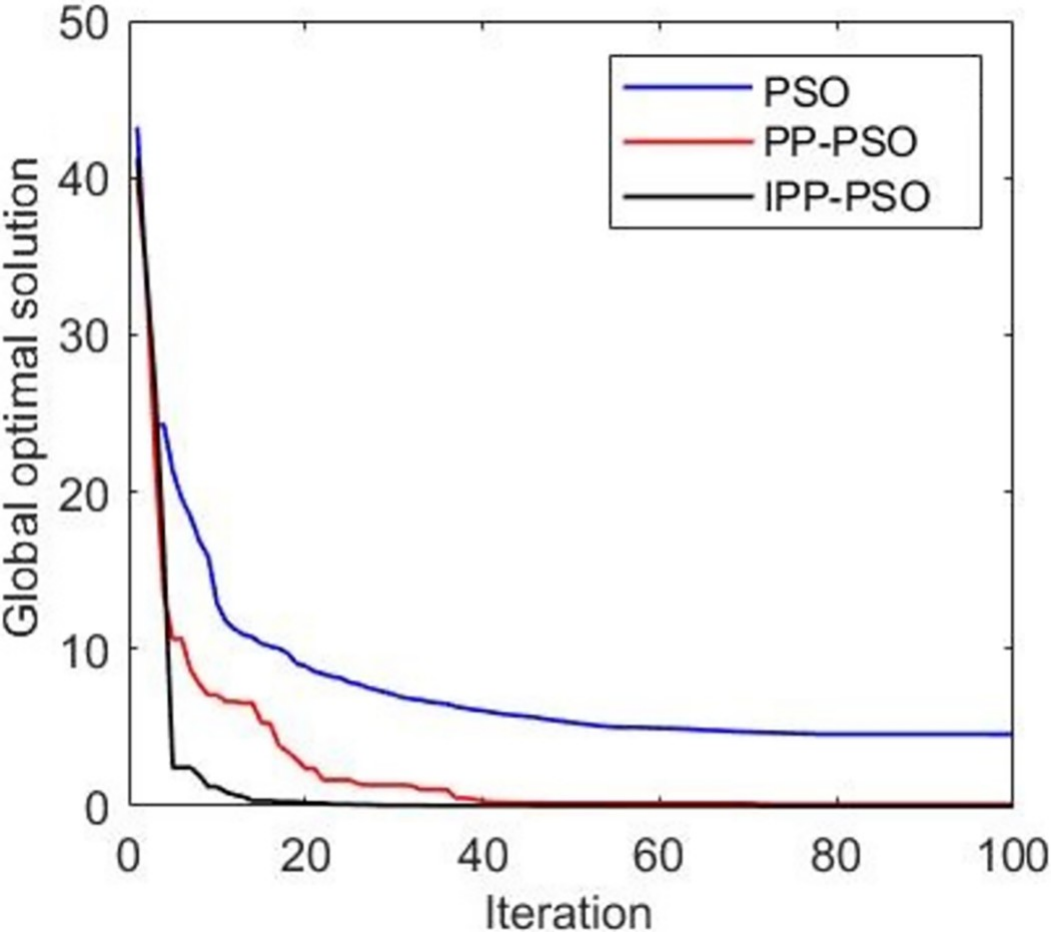
Figure of global optimal solution simulation results of two-person zero-sum game.

**Fig 6:**
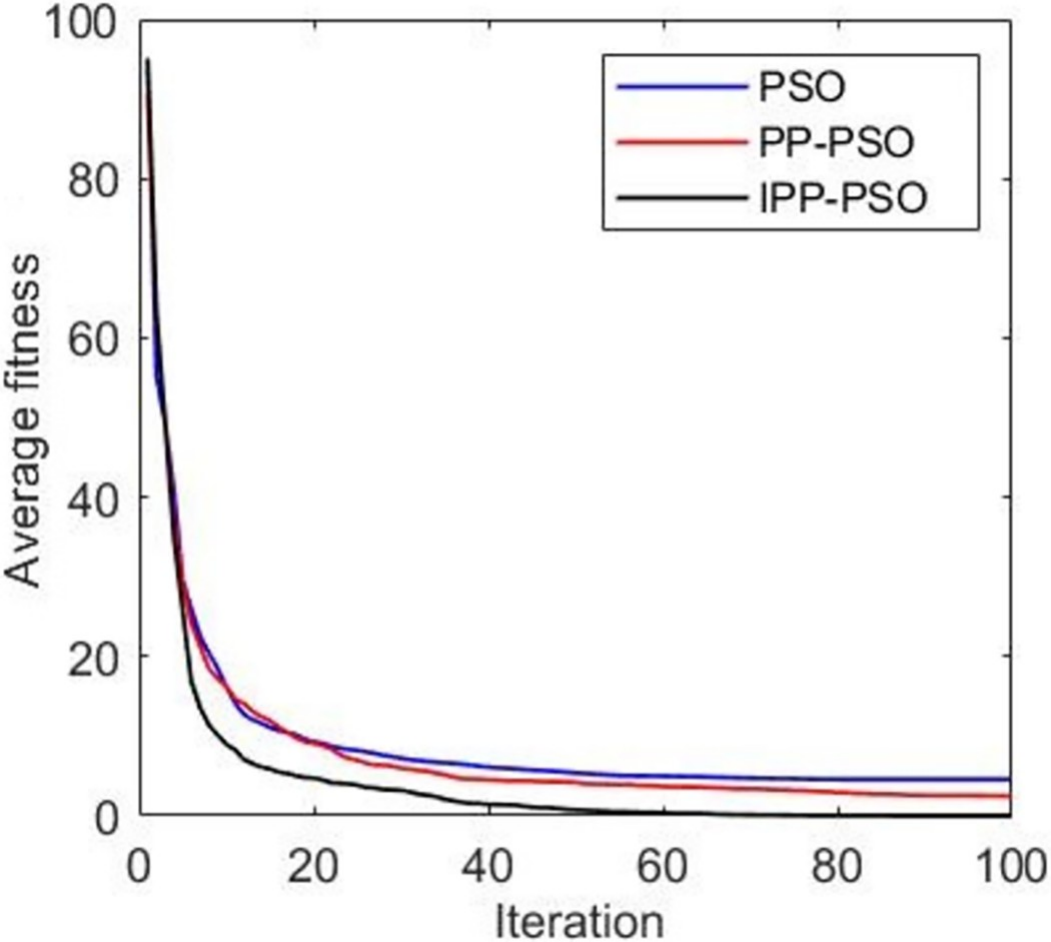
The average fitness function value of two—person zero—sum game simulation results.

**Fig 7:**
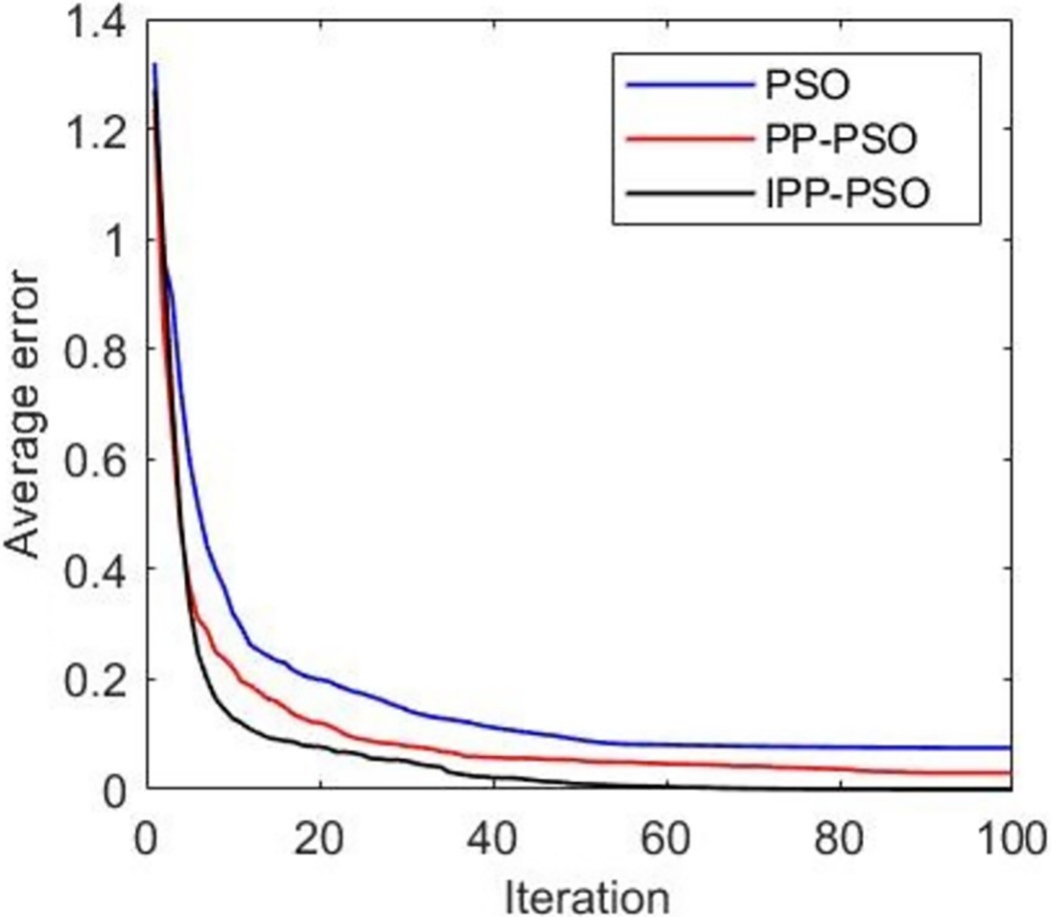
Average error simulation results of two-person zero-sum game.

In Tables 1 and 2, the first column and the first row represent the policies for Player 1 and Player 2, respectively. For example, in a zero-sum game, each player has three strategies that represent the number of rows and columns in the payoff matrix, respectively. Payments (or benefits) are set forth inside the table with the first number is the payout for the column player (Player 1), and the second is the payout for the row player (Player 2). To reduce statistical errors, each algorithm runs 100 independent tests on the two games. Figs 5 to 7 respectively present the simulation result curves of the three algorithms in terms of the global extreme value, the average fitness function value and the average error. Tables 3 and 4 show the average fitness function value, optimal fitness function value, mean minimum error, and the number of times of average error less than 0.0001 (the number of successes) of the three algorithms in 100 tests.

**Table 3:** Simulation results of two-person zero-sum game.

	Average fitness value	Optimal fitness value	Mean minimum error	Standard deviation	Number of successful
PSO	0.0675	4.8357e-8	0.0120	0.1279	4
PP-PSO	0.0128	5.9138e-4	0.0051	0.0731	0
IPP-PSO	0.0015	1.4794e-9	8.0634e-4	0.0047	97

**Table 4:** Simulation results of two-person non-zero-sum game.

	Average fitness value	Optimal fitness value	Mean minimum error	Standard deviation	Number of successful
PSO	8.2190	2.0876e-8	0.0210	0.2070	3
PP-PSO	0.0831	0.0056	2.6151e-3	0.0202	0
IPP-PSO	1.1077e-5	3.7714e-9	1.3308e-5	5.4336e-4	94

The definition of average error is shown in (34) and (35):

$$\begin{array}{c} e_{pp}\left(t\right)=\frac{\sum_{i=1}^{D}\left|\right|X_{di}\left(t\right)-E_{S}\left|\right|+\sum_{i=1}^{R}\left|\right|X_{ri}\left(t\right)-E_{S}\left|\right|}{D+R} \end{array}$$ (34)

$$\begin{array}{c} e_{p}\left(t\right)=\frac{\sum_{i=1}^{N}\left|\right|X_{i}\left(t\right)-E_{S}\left|\right|}{N} \end{array}$$ (35)

In (34), *e*_*pp*_(*t*) represents the average error of PP-PSO and IPP-PSO; in (35), *e*_*p*_(*t*) represents the average error of the original particle swarm optimization algorithm. *E*_*S*_ represents the mixed strategy Nash equilibrium solution. The *E*_*S*_ in Table 1 is:

$$\begin{array}{c} E_{S}=\left[\frac{21}{52},\frac{12}{52},\frac{19}{52},\frac{2}{13},\frac{3}{13},\frac{8}{13}\right] \end{array}$$ (36)

The *E*_*S*_ in Table 2 is:

$$\begin{array}{c} E_{S}=\left[\frac{1}{3},\frac{1}{3},\frac{1}{3},0,\frac{1}{3},\frac{1}{3},\frac{1}{3},0\right] \end{array}$$ (37)

Fig 5 shows that the global optimal solution is the global optimal fitness function value, and the smaller the fitness value, the better the result. IPP-PSO approximately converges to the global optimal solution after about 20 iterations, PP-PSO approximately converges to the global optimal solution after about 40 iterations, and the PSO algorithm does not converge to the global optimal solution in this simulation. The convergence speed of IPP-PSO algorithm is faster than that of PP-PSO and PSO algorithm, and it can converge to the global optimal solution. Fig 6 shows the change of the average fitness function value with the number of iterations. The average fitness value represents the average level of fitness values of all particles in the algorithm. It can be seen from Fig 6 that the IPP-PSO algorithm improves the problem that the average fitness function value of PP-PSO algorithm cannot converge, and the convergence speed is better than that of the PSO algorithm. Fig 7 shows the variation of the average error with the number of iterations. The PP-PSO algorithm and PSO algorithm fail to converge near the global optimum, and the average error is large. The improved IPP-PSO algorithm improves this problem, and the “precocity” problem of the algorithm is improved because pathfinder is added to improve the population diversity. This addition also speeds up the convergence speed of the algorithm.

Tables 3 and 4 show the simulation results of 100 tests, which reduces the contingency of the algorithm in a simulation and can better show the real performance of the algorithm. The average fitness value is the average of the optimal fitness value of each test, the optimal fitness value is the minimum fitness value of the 100 tests, the mean minimum error is the average of the mean minimum error of each test, and the number of successes is the number of times the mean error is less than 0.0001. By comparing the three algorithms in Tables 3 and 4, it can be concluded that the IPP-PSO algorithm has the minimum average fitness value, minimum average error, minimum optimal fitness value and the most successful times, which further demonstrates that the IPP-PSO algorithm has better performance than both the PP-PSO algorithm and the PSO algorithm in solving Nash equilibrium problems.

According to the above analysis, the IPP-PSO algorithm is superior to the PP-PSO algorithm and the original PSO algorithm in terms of solving accuracy, convergence speed and reliability of Nash equilibrium calculation. It can be used to solve the Nash equilibrium of the game problem of attack and defense confrontation of clustered UAVs.

## Conclusion

When we study the game model of attack and defense confrontation of swarm UAVs, we encounter the problem of choosing the best algorithm to solve the game model. In the process of trying to solve Nash equilibrium with PSO and PP-PSO, combined with the results of previous studies, we find the convergence and local optimality of these two algorithms. An IPP-PSO algorithm is proposed to solve the Nash equilibrium of the game. By improving the distribution of predator and prey, the formula of inertia weight and velocity of predator and prey particles, and adding pathfinder particles, the algorithm improves the problems of the original algorithm, such as “precocity”, slow convergence and local optimum. The performance of PSO, PP-PSO and IPP-PSO algorithm in solving Nash equilibrium of a game is compared with simulation examples. Simulation results show that the IPP-PSO algorithm is convergent and effective, and the convergence speed and global optimal performance are better than the other two algorithms. The improved algorithm can be used to solve the game model of attack and defense confrontation of clustered UAVs.

## Supporting information

S1 Data (XLSX)

S2 Data (XLSX)

S3 Data (XLSX)

S4 Data (XLSX)

S5 Data (XLSX)

S6 Data (XLSX)

S7 Data (XLSX)

S8 Data (XLSX)

S9 Data (XLSX)

S10 Data (XLSX)

S11 Data (XLSX)

S12 Data (XLSX)

S13 Data (XLSX)

S14 Data (XLSX)

S15 Data (XLSX)

S16 Data (XLSX)

S17 Data (XLSX)

S18 Data (XLSX)
